# The Role of Speciation in Positive Lowenstein-Jensen Culture Isolates from a High Tuberculosis Burden Country

**DOI:** 10.1371/journal.pone.0027017

**Published:** 2011-11-04

**Authors:** William Worodria, Jillian Anderson, Adithya Cattamanchi, J. Lucian Davis, Saskia den Boon, Alfred Andama, Samuel D. Yoo, Moses Joloba, Laurence Huang, Midori Kato-Maeda

**Affiliations:** 1 Department of Medicine, Makerere University College of Health Sciences, Kampala, Uganda; 2 Department of Medicine, Mulago National Referral and Teaching Hospital, Kampala, Uganda; 3 San Francisco Research Collaboration, Makerere University-University of California San Francisco, Kampala, Uganda; 4 Division of Pulmonary and Critical Care Medicine, Department of Medicine, University of California San Francisco, San Francisco General Hospital, San Francisco, California, United States of America; 5 Curry International Tuberculosis Center, Department of Medicine, University of California San Francisco, San Francisco General Hospital, San Francisco, California, United States of America; 6 HIV/AIDS Division, Department of Medicine, University of California San Francisco, San Francisco General Hospital, San Francisco, California, United States of America; 7 Department of Microbiology, Makerere University School of Biomedical Sciences, Kampala, Uganda; University of Stellenbosch, South Africa

## Abstract

**Objective:**

To determine the need for routine speciation of positive Lowenstein-Jensen mycobacterial cultures in HIV-infected patients suspected of having pulmonary tuberculosis at Mulago Hospital in Kampala, Uganda.

**Methods:**

Sputum and bronchoalveolar lavage Lowenstein-Jensen mycobacterial culture isolates from consecutive, HIV-infected patients admitted to Mulago Hospital with 2 weeks or more of cough were subjected to IS*6110* PCR and *rpoB* genetic analysis to determine the presence of *Mycobacterium tuberculosis* complex (MTBC) and non-tuberculous mycobacteria (NTM).

**Results:**

Eighty (100%) mycobacterial cultures from 65 patients were confirmed to be members of MTBC. Subsequent analysis of the cultures from 54 patients by PCR and sequence analyses to identify co-infection with NTM confirmed the presence of MTBC as well as the presence of *Micrococcus luteus* (n = 4), *Janibacter spp.* (n = 1) and six cultures had organisms that could not be identified.

**Conclusions:**

Presumptive diagnosis of tuberculosis on the basis of a positive Lowenstein-Jensen culture is sufficient in HIV-infected Ugandans suspected of having tuberculosis. Routine molecular confirmation of positive Lowenstein-Jensen cultures is unnecessary in this low resource setting.

## Introduction

In sub-Saharan Africa, patients with cough greater than two weeks who have positive sputum acid-fast bacilli (AFB) smear examinations or positive sputum mycobacterial cultures are usually diagnosed with pulmonary tuberculosis (TB). Speciation of culture isolates by nucleic acid amplification or biochemical techniques is rarely performed. However, pulmonary disease may also be due to non-tuberculous mycobacteria (NTM), particularly in HIV-infected patients. Several recent studies from sub-Saharan Africa suggest the prevalence of NTM disease may be higher than previously recognized [1]–[4]. One study has also reported isolating NTM species from water and soil in Uganda [5]. Given the increased risk of NTM lung disease in HIV-infected patients and the possibility of culture contamination by environmental mycobacteria, rapid speciation assays that distinguish NTM from *Mycobacterium tuberculosis* complex (MTBC) could improve the specificity of mycobacterial culture for TB diagnosis. However, such assays increase costs and increase the workload of already over-extended mycobacterial laboratories in high-burden countries. To determine whether routine speciation of positive Lowenstein-Jensen (LJ) cultures is necessary in a population at high risk for NTM disease, we performed PCR targeting the insertion sequence (IS) *6110* and RNA polymerase β-subunit-encoding (*rpoB*) genes on all available mycobacterial cultures collected from a hospital-based sample of HIV-infected patients with TB. [6]–[8]

## Methods

### Study Population and Patient Evaluation

We included all mycobacterial cultures isolated between April and August 2007 from a hospital-based study of HIV-infected patients. Briefly, consecutive HIV-infected adults admitted to the medical wards of Mulago Hospital in Kampala with cough of two weeks or more were included in the study. All individuals submitted one spot and one early morning sputum specimen for AFB smear and mycobacterial culture. If initial sputum smears were AFB negative, patients were referred for bronchoscopy with airway inspection for tracheobronchial lesions of Kaposi's sarcoma and bronchoalveolar lavage (BAL) fluid examination for mycobacteria (AFB smear and culture), *Pneumocystis jirovecii* (modified Giemsa stain), and other fungi (potassium hydroxide smear and culture).

### Specimen Processing

All sputum and BAL fluid specimens were processed at the Uganda National Tuberculosis Reference Laboratory (NTRL) using standardized methods for digestion and decontaminated using 1% N-acetyl-L-cysteine (NALC), 2% sodium hydroxide (NaOH), and 2% sodium citrate solution. The resulting pellets were inoculated on two LJ slants per sample and incubated at 37°C. NTRL technicians stored all cultures positive for mycobacteria (confirmed by Ziehl-Neelsen staining) in liquid culture media (equal proportions of 25% glycerol and Middlebrook 7H9) and stored them at −80°C until shipping. A subset of 57 cultures that was successfully stored was sent to the *Mycobacterium tuberculosis* Research Laboratory (MTBRL) at the University of California San Francisco for PCR-based and gene-sequence analyses. At MTBRL, cells were sub-cultured in LJ slants, Middlebrook 7H9 broth, and/or Middlebrook 7H11 agar, and incubated at 37°C. DNA was extracted using a standardized method of heat inactivation and/or cetyl-trimethylammonium bromide (CTAB)/Chloroform Isoamyl Alcohol [9]–[10].

### Identification of MTB complex

Initial speciation of mycobacterial culture isolates was done using IS*6110* PCR at the Makerere University Department of Microbiology. Briefly, DNA was extracted from thawed frozen culture isolates using thermolysis. IS*6110* PCR was performed using primers described by Muhumuza *et al.* [11]. IS*6110* is a non-coding region specific for MTB, and the presence of a 521-base-pair band in the gel electrophoresis was considered compatible with MTBC. For a subset of 57 isolates available for additional analyses, IS*6110* PCR analysis using a second set of primers [10] was performed at the MTBRL. The presence of a 245 base-pair fragment was considered positive for MTBC.

### Identification of NTM

We used primer-specific amplification within the *rpoB* gene (primers have a sequence unique for either MTBC or NTM at the 3′ end of each primer) in 55 of the 57 cultures sent to the MTBRL to differentiate between MTBC and NTM [7]. *rpoB* encodes the RNA polymerase β-subunit and contains single nucleotide polymorphisms that can be used to differentiate among mycobacterial species. The primers for MTBC were Tbc1 (5′-CGTACGGTCGGGGAGCTGATCCAA-3′) and TbcR5 (5′-CCACCAGTCGGCGCTTGTGGGTCAA-3′), and the primers for NTM were M5 (5′-GGAGCGGATGACCACCCAGGACGTC-3′) and RM3 (5′-CAGCGGGTTGTTCTGGTCCATGAAC-3′). Briefly, 10 pM of Tbc1 and TbcR5 primers, 20 pM of M5 and RM3 primers and 1–5 µl of DNA were added to FailSafe PCR 2× PreMix B (Epicentre, Madison, WI) and/or AccuPower PCR PreMix (Bioneer, Daejeon, Korea) and PCR was performed with the following parameters: initial denaturation at 95°C for 5 min, 30 cycles of amplification (at 95°C for 30 sec, then at 72°C for 60 sec), and final elongation at 72°C for 5 min. PCR products were analyzed by agarose gel electrophoresis (1%). In all PCR reactions, *Mycobacterium tuberculosis* (*M.TB)* H37Rv DNA was used as a positive control for MTBC, and *M. fortuitum* (ATCC 6841) or *M. kansasii* (ATCC 12478) DNA was used as a positive control for NTM.

PCR products with a size of 136 bp (suggestive of NTM) were purified and sequenced to confirm and identify the species of NTM. The sequence reaction was performed using NTM-specific primers RM3 and M5, and products were sequenced at the UCSF Genomic Core Facility using ABI Big-Dye v3.1 dye terminator sequencing chemistry (Applied Biosystems, Carlsbad, CA) and the ABI PRISM 3730×l capillary DNA analyzer. Sequence data was analyzed using ApE v1.17 and basic local alignment search was performed using National Center for Biotechnology Information (NCBI)'s genomic Basic Local Alignment Search Tool (BLAST) (http://blast.ncbi.nlm.nih.gov/Blast.cgi).

### Microscopy

Kinyoun and Gram stain (BD, Franklin Lakes, NJ) slides were prepared for isolates that were *rpoB* PCR positive for the 136-bp fragment (suggestive of NTM).

### Definitions

We considered mycobacterial cultures to contain MTBC if the IS*6110* PCR resulted in a product compatible with an IS*6110* element or if *rpoB* PCR resulted in a product of 235 bp. We considered mycobacterial cultures to contain NTM if *rpoB* PCR resulted in a product of 136 bp and the sequence analysis results were compatible with NTM species. If *rpoB* PCR resulted in a 136-bp product but sequencing results were unavailable or inconclusive, or if direct microscopy showed multiple organisms including AFB, we considered mycobacterial cultures to possibly contain NTM.

### Statistical analysis

We summarized demographic characteristics of the study population as medians and frequencies, and compared demographic and clinical characteristics using Fisher's exact test for categorical variables and Mann-Whitney rank-sum test for continuous variables. We defined our threshold for statistical significance as p<0.05. We used STATA 10.0 (Stata Corporation, College Station, Texas, USA) for all statistical analyses.

### Ethical considerations

The Makerere University Faculty of Medicine Research Ethics Committee, the Mulago Hospital Institutional Review Board, the Committee on Human Research at the University of California, San Francisco Committee on Human Research, and the Uganda National Council for Science and Technology approved the study protocol. All study participants provided written informed consent.

## Results

### Study population

**Figure 1:**
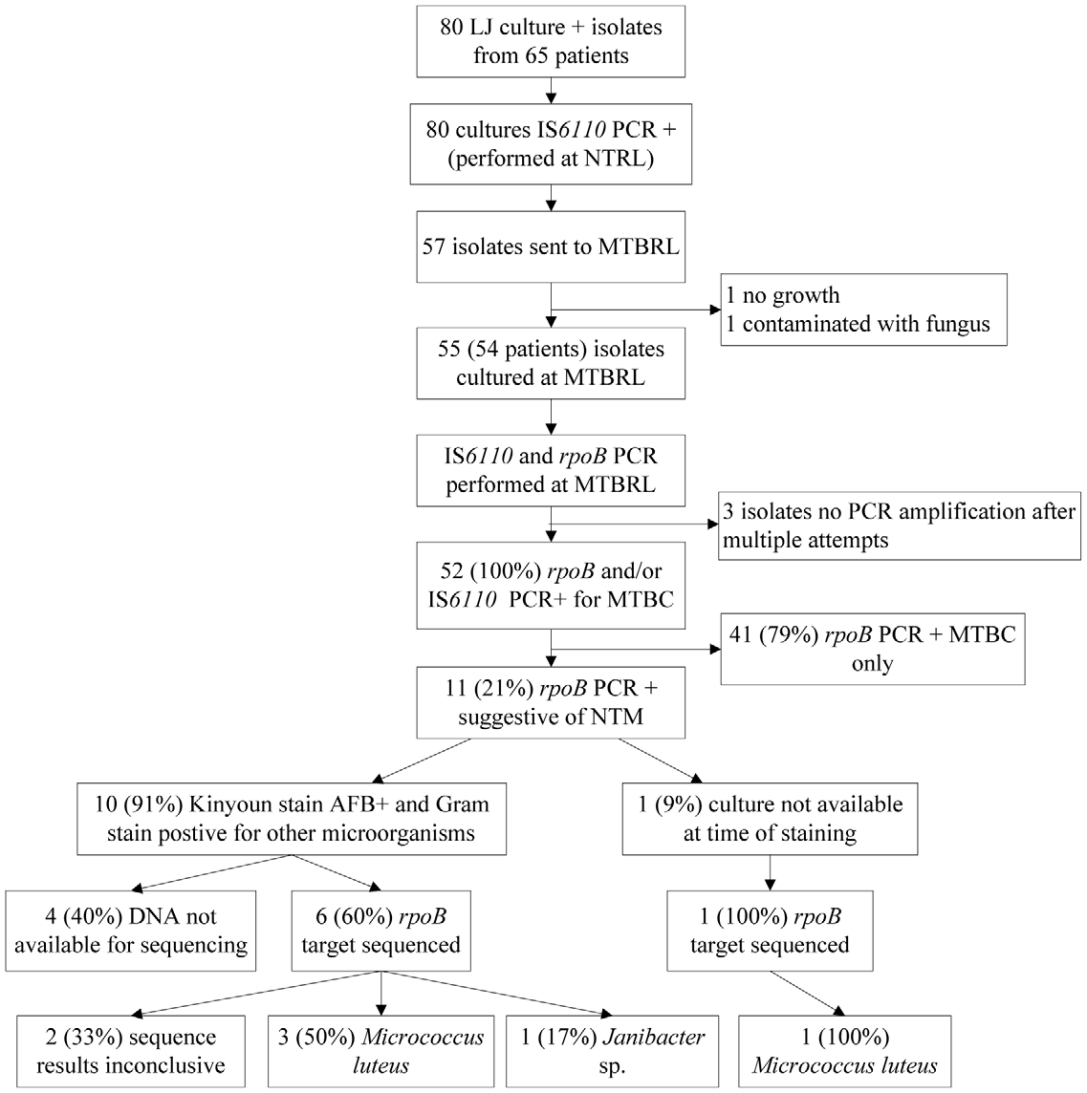
PCR and sequencing results to determine the presence of NTM. Of the 52 isolates with valid PCR results, all were positive for MTBC and 11 (21%) isolates had an *rpoB* PCR product suggestive of NTM. The sequence result showed that 4 of the 11 were compatible with *Micrococcus luteus* and 1 with *Janibacter sp*, the remaining 6 (55%) did not have sequence results available. No NTM species were found.

We included 80 mycobacterial cultures from 65 consecutive HIV-positive patients (Figure 1.), with median CD4+ T-lymphocyte count of 30 cells/µL (inter-quartile range (IQR), 10-106 cells/µL). Patients were predominantly male (54%), and the median age was 33 years (IQR, 28–39 years). Of the 80 culture isolates, 75 were obtained from sputum and 5 from BAL fluid. There were no significant differences in demographic or clinical characteristics between the 54 patients who had mycobacterial culture isolates (N = 55 isolates) tested at MTBRL and the 11 patients for whom mycobacterial culture isolates (N = 25 isolates) were unavailable for further analysis.

### MTBC detection

*IS6110* PCR performed at the Makerere University Department of Microbiology resulted in a 521 base-pair product in all 80 isolates, indicating that all the cultures contained MTBC. Of the 80 culture isolates, 57 were available for further analysis at MTBRL and 55 were successfully sub-cultured. The presence of MTBC was confirmed in 52 (95%) of 55 cultures either by amplification of the IS*6110* product (50 isolates), and/or by amplification of a 235-bp product from the *rpoB* gene target (34 isolates) (Figure 1.). In three cultures, PCR amplification did not produce any product of any size despite several attempts.

### NTM detection

Of the 52 cultures with MTBC, 11 also had an *rpoB* PCR product of 136-bp suggestive of NTM. Smears were prepared from 10 of the 11 cultures for microscopic examination (the culture for one was not available at the time of the slide preparation). Kinyoun staining showed that all 10 cultures contained AFB-positive organisms as well as other organisms (Table 1.). Sequencing of the *rpoB* gene showed 4 matches with *Micrococcus luteus*, 1 with *Janibacter spp.* (Figure 1. Table 1.), and two that did not match with any organism, probably due to the presence of two or more organisms. The other 4 isolates showed products of 136 bp and 235 bp in the initial PCR suggesting the presence of NTM and MTBC (the negative control of the PCR showed no contamination). However, when PCR was repeated for sequencing analysis, we were not able to amplify the 136 bp product, possibly due to the low concentration of 136 bp specific DNA relative to the 235 bp specific DNA, despite multiple attempts.

**Table 1:** Molecular and microscopy results for the 11 isolates with MTBC and other organisms.

Isolate	*rpoB* PCR	*IS*6110 PCR	Sequence results	Kinyoun stain	Gram stain
1	136 bp and 235 bp	+	*Janibacter sp*.	AFB+	Gram+ bacilli
2	136 bp	+	*M. luteus*	AFB+	Gram+ bacilli
3	136 bp	+	*M. luteus*	AFB+	Gram+ bacilli
4	136 bp and 235 bp	+	*M. luteus*	AFB+	Gram+ bacilli
5	136 bp and 235 bp	+	N/A	AFB+	Gram+ cocci
6	136 bp and 235 bp	+	N/A	AFB+	Gram+ bacilli and cocci Gram- cocci
7	136 bp and 235 bp	+	N/A	AFB+	Gram− bacilli
8	136 bp and 235 bp	+	N/A	AFB+	Gram− bacilli, Gram variable bacilli
9	136 bp and 235 bp	+	N/A	AFB+	Gram+ bacilli
10	136 bp and 235 bp	+	N/A	AFB+	Gram+ cocci, Gram- cocci
11	136 bp and 235 bp	+	*M. luteus*	N/A	N/A

### Clinical outcomes

The 11 cultures shown to have MTBC plus other organisms belonged to 11 patients. The demographic and clinical characteristics of these patients were similar to the 41 patients who had a pure culture of MTBC (Table 2.). Of the 52 patients, 49 (94%) had vital status ascertained two months after admission: 10 of 11 from the patients who had MTBC plus other organisms, and 39 of 41 from the patients with pure MTBC growth. Survival at 2 months was similar among these two groups of patients. Death occurred in 2/10 (20%) of the patients with a growth of MTBC plus other organisms and in 8/39 (21%) patients with pure MTBC growth.

**Table 2:** Demographic and Clinical Characteristics of patients by culture growth of MTB with or without other organisms.

Characteristic	MTB only	MTB plus other growth	p-value
	(N = 41)	(N = 11)	
Female (%)	19 (46)	6 (55)	0.74
Median age (IQR)	32 (28–39)	36 (31–39)	0.38
Current/Prior Smoker (%)	14 (34)	4 (36)	1.00
Symptoms			
Fever (%)	40 (98)	11 (100)	1.00
Hemoptysis* (%)	7 (18)	0(0)	0.58
Weight loss (%)	39 (95)	9 (82)	0.19
Shortness of breath (%)	24 (59)	8 (73)	0.50
Cough (%)	41 (100)	11 (100)	-
Chest pain (%)	24 (59)	7(64)	1.00
Anti-retroviral therapy* (%)	6 (24)	1 (13)	0.65
Co-trimoxazole prophylaxis* (%)	22 (88)	7 (88)	1.00
Median CD4 count* (IQR)	48 (12–152)	15 (9–68)	0.14

*Had missing values.

## Discussion

In this study, we examined the specificity of Lowenstein-Jensen culture for the diagnosis of TB using respiratory specimens obtained from hospitalized, HIV-infected patients in Uganda suspected to have pulmonary TB. We found that the 80 cultures that grew mycobacteria all had positive molecular tests for MTBC (100% specificity of LJ cultures for diagnosing TB). Interestingly, further examination of 11 of 52 cultures showed the presence of another organism, either by molecular techniques including PCR and sequencing, or by smear examination. In six cultures, we could not definitively rule out the presence of NTM; however, all had MTBC. This finding suggests that routine molecular testing of positive LJ cultures is not needed to define the presence of MTBC, because all of the patients in this sample of HIV patients with cough of two weeks or more seeking medical attention in Mulago Hospital had TB.

Rapid speciation assays to confirm the presence of MTBC and rule out NTM have been recently introduced. Despite limited data, the WHO has recommended that such assays be routinely used to confirm the presence of MTBC in all positive results obtained from liquid culture systems [12]. One such rapid test is the Capilia TB assay, a lateral-flow immunochromatographic assay that detects the MPT64 antigen in MTBC culture isolates. This test is comparable in performance to conventional biochemical assays for confirmation of MTBC and significantly cheaper [13]. Previous studies from Africa reporting isolation of NTM species from respiratory specimens have shown variable results. An early study from Uganda reported the absence of NTM disease in blood cultures of severely ill Ugandan HIV-infected patients with AIDS [14]. However, in other parts of the continent, the frequency of NTM isolation is considerably higher. In Ghana, 23% of patients with bronchopulmonary symptoms longer than four weeks had NTM isolated from LJ cultures of respiratory specimens [15]. However, the HIV serostatus of these patients was not reported while colony appearances and biochemical tests were used to identify NTM. Similarly, studies from South Africa describe NTM as a frequent isolate from LJ cultures of sputum samples from miners (incidence 47.6 per 100,000 person-years) and from HIV-infected populations [2]. In a study from Mozambique, Nunes *et al.* found NTM in 3 (1%) out of 277 isolates grown from LJ cultures of sputum or BAL samples of patients who were HIV positive [16]. The frequency of patients with cultures positive for both *M.TB* and NTM varies also depending on the population and the region studied. In India, 2% of 6,143 AFB-positive cultures were identified as NTM based on speciation by *rpoB* sequencing; 6 of these were found to be pulmonary cases of mixed NTM and *M.TB* infection [17]. A study of clinical TB suspects in and around New Delhi found *M. avium* and *M.TB* co-infection in 2.9% of an HIV-infected study population [18]. In Zambia, the frequency of positive liquid cultures for *M.TB* and NTM varied between 6 and 14% among a hospital-based population of chronically ill adult patients, 75% of them infected with HIV [19]. Thirty-one patients (17%) had NTM isolated; of these, only one patient fulfilled the American Thoracic Society criteria for NTM lung disease [19]. Based on these data, it is evident that the frequency of NTM varies depending on the geographic location and local epidemiology of NTM disease.

In regions where NTM prevalence is known to be high reliance on ZN staining alone to identify MTB from growth on solid culture media may lead to overtreatment of patients. It may also lead to development of resistance since many NTM are partially but incompletely responsive to standard MTB treatment.

### Other organisms identified

Two of the organisms we identified have been infrequently reported as causes of human disease. *M. luteus* is a commensal, non-pathogenic organism found on mammalian skin and in soil and environmental water sources. *M. luteus* infections are rare but have been reported in patients with in-dwelling plastic medical devices; they have also been reported in association with folliculitis in patients with HIV-1 disease [20]. The *Janibacter* species sequenced in this study most closely matched a recently described marine *Janibacter* strain and aside from one case report involving an immunocompromised host with an indwelling central venous catheter, all isolation of *Janibacter* species has been from environmental sources [21]–[22]. Given the clinical presentations of the patients in this study and the generally non-pathogenic nature of the two environmentally occurring microbes, it is likely that *M. luteus* and *Janibacter spp*. cultured in this study are due to contamination.

Our study has some limitations. First, we used a convenience sample of HIV-infected patients receiving care in Mulago Hospital in Kampala, which introduces potential bias as our sample may not be representative of the general HIV-infected population. Therefore, our findings may not be applicable to other populations and geographic regions. Second, the initial cultures for mycobacteria were performed only on solid media, which has a low recovery rate for NTM [23], but liquid culture systems were unfortunately not in routine use at the Uganda NTRL at the time of the study. Additional studies evaluating relevant populations of tuberculosis suspects would help guide practice in laboratories using liquid culture systems. Third, we cannot exclude the possibility of co-infection with MTBC and NTM species in the 6 (11.5%) of 52 cultures in which the sequence result was not available.

In summary, all of the patients in this study of HIV-infected patients with cough of two weeks or more with a Lowenstein-Jensen culture positive for mycobacteria in respiratory specimens had tuberculosis. This finding suggests that routine speciation is unnecessary in HIV-infected patients admitted to Mulago Hospital with suspected pulmonary TB and positive LJ cultures. Future studies using liquid culture systems and NTM-specific PCR assays should be conducted to confirm our findings and to evaluate further for the possibility that mixed infections are present.
